# Supplementary figures and images for: The ROP16III-dependent early immune response determines the subacute CNS immune response and type III Toxoplasma gondii survival
Source: PLoS Pathog. 2019 Oct 24;15(10):e1007856. doi: 10.1371/journal.ppat.1007856 (PMC6812932; doi:10.1371/journal.ppat.1007856)

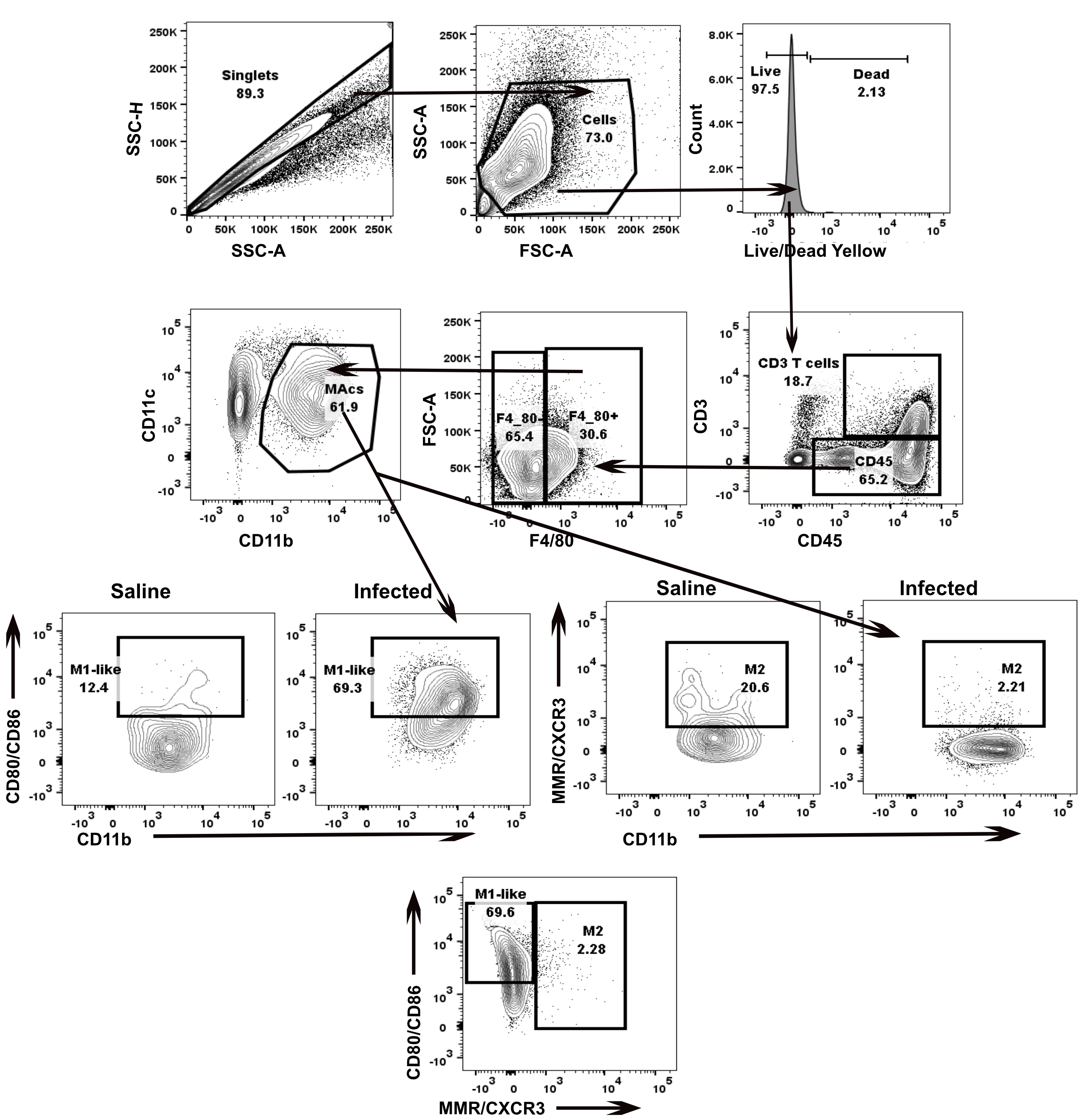

Supplement: S1 Fig — Immune cells were isolated from the brain and stained for macrophage markers. Single cells were discriminated from doublets by plotting side scatter height (SSC-H) versus side scatter area (SSC-A). Cells were selected by plotting SSC-A versus forward scatter area (FSC-A). Live cells were gated on live/dead Yellow-. CD45+ CD3- cells were gated by plotting CD3 versus CD45. From the CD45+ gate, F4/80+ and F4/80- cells were gated by plotting FSC-A versus F4/80. From the F4/80+ gate, macrophages (Macs) were gated by plotting CD11c versus CD11b. From the Macs gate, (CD80+/CD86+) M1-like macrophages were gated by plotting CD80/CD86 versus CD11b. From the Macs gate, (MMR+/CxCR3+) M2 macrophages were gated by plotting MMR/CXCR3 versus CD11b. Uninfected controls and isotype controls were used to establish the gating scheme. The last image shows CD80/CD86 versus MMR/CxCR3. The gates for the M1-like and M2 populations were determined by the values for CD80+/CD86+ cells and MMR+/CxCR3+ cells in the preceding analysis. (TIF) [file ppat.1007856.s001.tif]

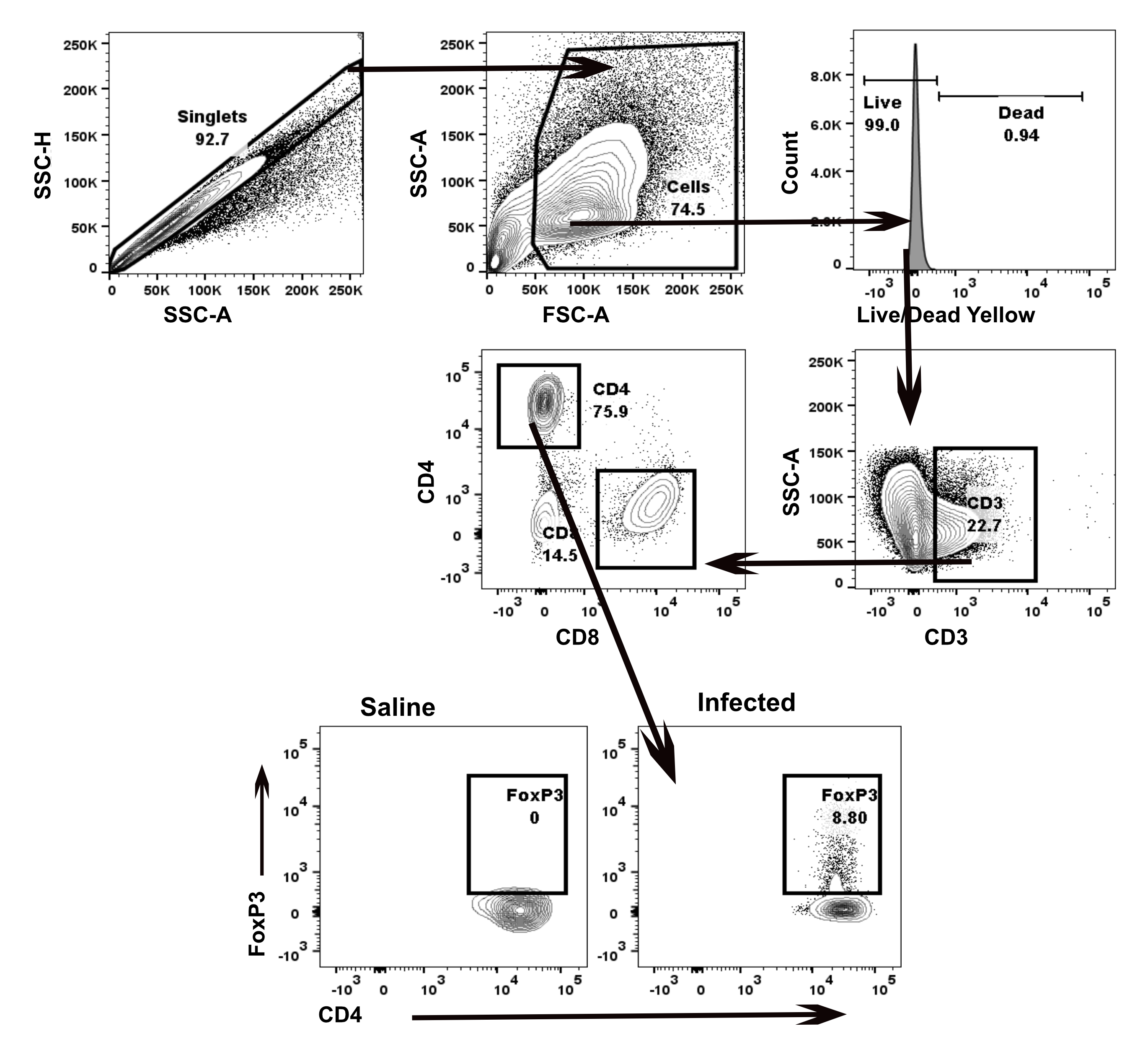

Supplement: S2 Fig — Immune cells were isolated from the brain and stained for T cell markers. Single cells were discriminated from doublets by plotting side scatter height (SSC-H) versus side scatter area (SSC-A). Cells were selected by plotting SSC-A versus forward scatter area (FSC-A). Live cells were gated on live/dead Yellow-. CD3+ cells were gated by plotting SSC-A versus CD3. From the CD3+ gate, CD4+ and CD8+ cells were gated by plotting CD4 versus CD8. From the CD4+ gate, FoxP3+ Tregs were gated by plotting FoxP3 versus CD4. Uninfected controls and isotype controls were used to establish the gating scheme. (TIFF) [file ppat.1007856.s002.tiff]

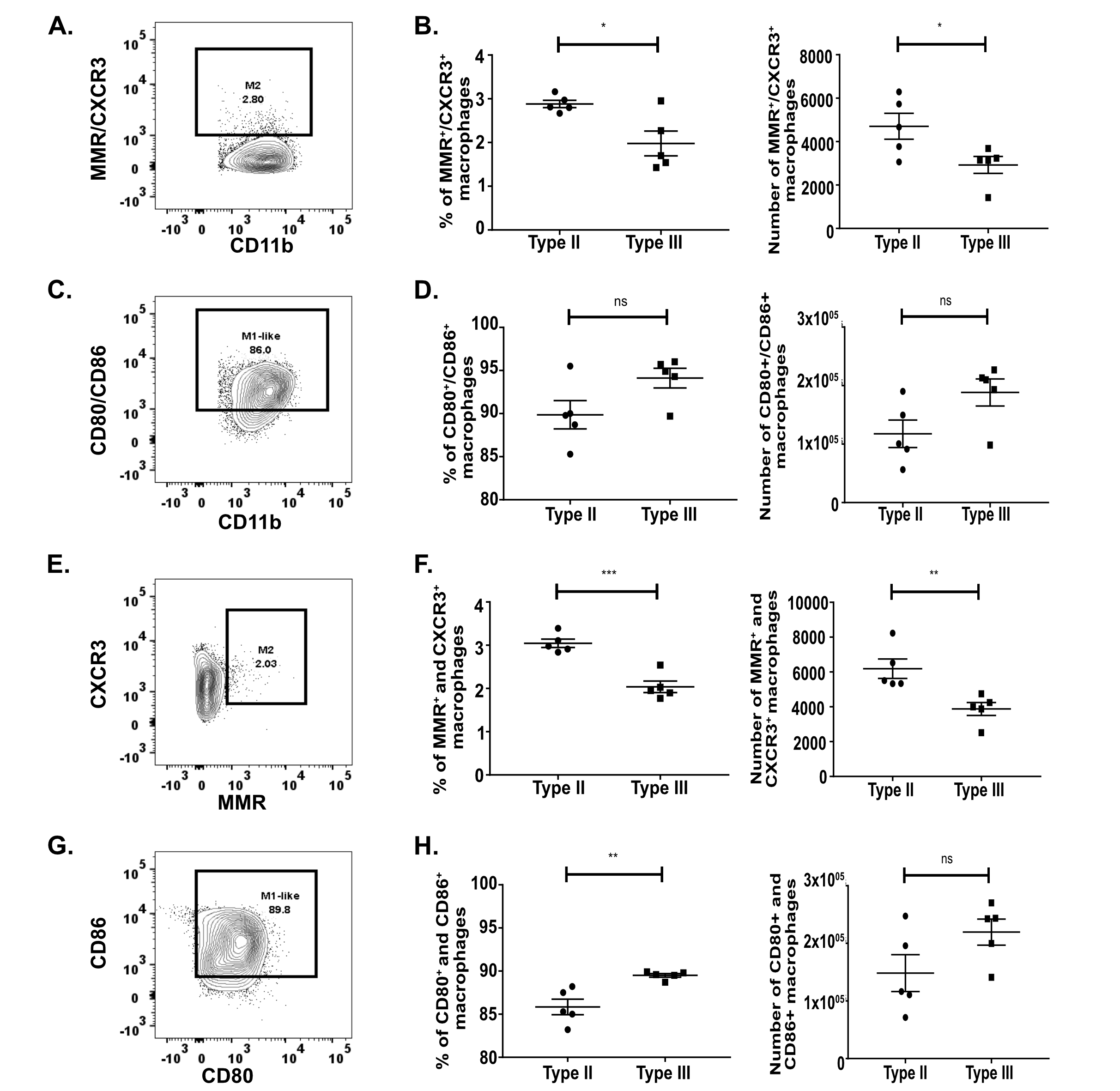

Supplement: S3 Fig — At 21 dpi, immune cells were isolated from the CNS of either type II- or type III-infected mice, split, stained for macrophage markers, and then analyzed by flow cytometry. A,B. For type II-infected mice, the percentage and number of M2 macrophages identified by placing MMR and CXCR3 in the same channel or separate channels. C,D. For type II infected mice, the percentage and number of M1-like macrophages identified by placing CD80 and CD86 in the same channel or separate channels. E.F. As in (A,B) except for type III-infected mice. G,H. As in (C,D) except for type III-infected mice. Bars, mean ± SEM. N = 5 mice/infected group. ns = not significant, non-parametric t-test. (TIF) [file ppat.1007856.s003.tif]

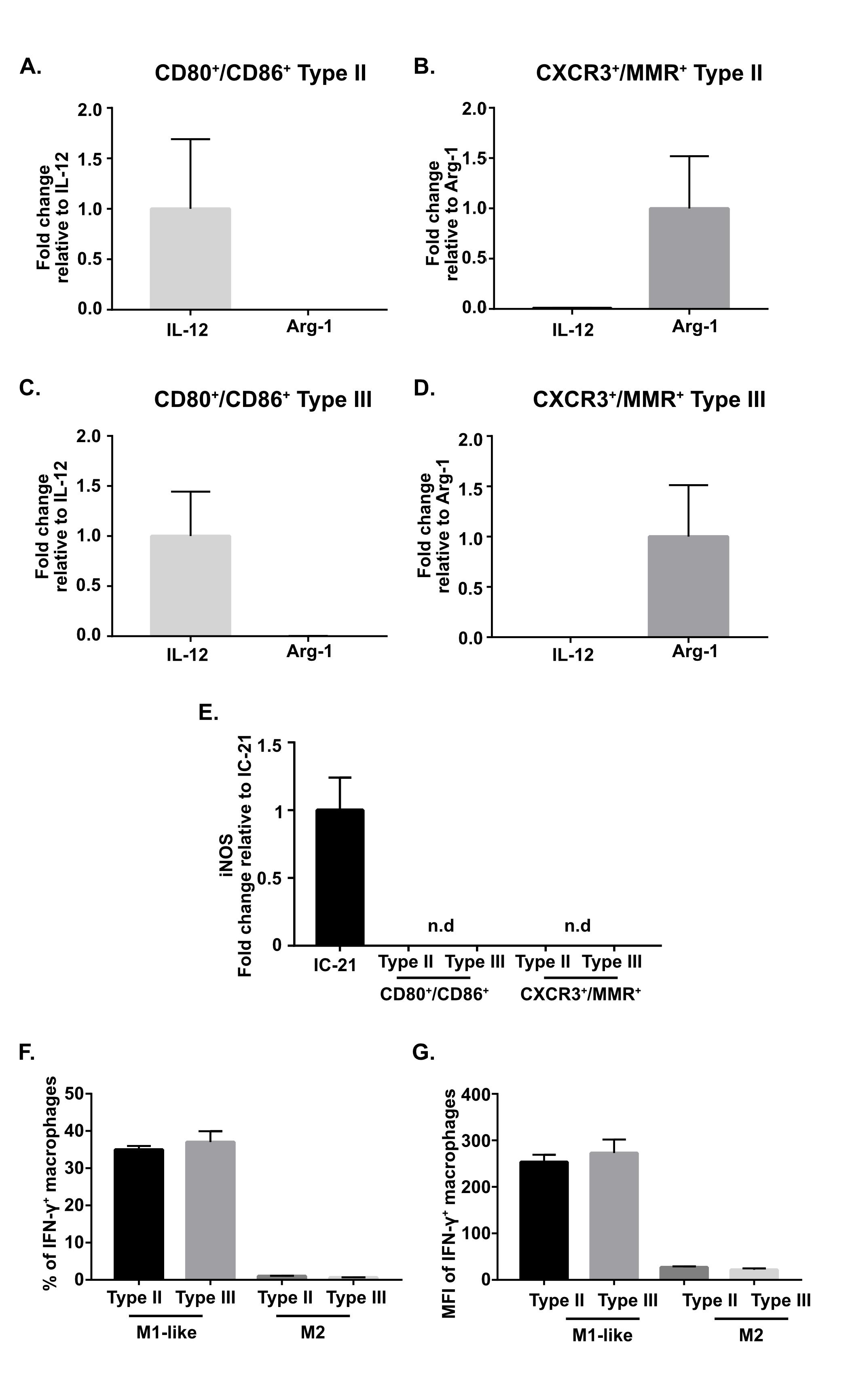

Supplement: S4 Fig — Mice were inoculated with type II or type III parasites. A,B. At 5dpi, splenocytes were isolated, stained, and sorted into M1-like macrophages and M2s. Q-PCR was performed on RNA isolated from these cells. Graphs show Q-PCR quantification of IL-12, Arg-1 expression from M1-like macrophages and M2s from type II-infected mice. C,D. As in (A,B) except from M1-like macrophages and M2s from type III-infected mice. E. As in (A,B) except for iNOS and using IFN-γ and LPS-stimulated IC-21 cells (macrophage cell line) as a positive control. iNOS is listed as nd (not detected) in the samples from infected mice because melting curve analysis and gel electrophoresis showed no product in these reactions. N = 5 Mice/infected group. F,G. M1-like macrophages and M2s isolated from the brain of 3 wpi mice were analyzed for cellular IFN-γ production by flow cytometry. F. Frequency of IFN-γ producing M1-like macrophages or IFN-γ producing M2s. G. Quantification of the mean fluorescent intensity of IFN-γ in M1-like macrophages and M2s. N = 6 mice/infected group. A-G, bars = mean ± SEM. (TIF) [file ppat.1007856.s004.tif]

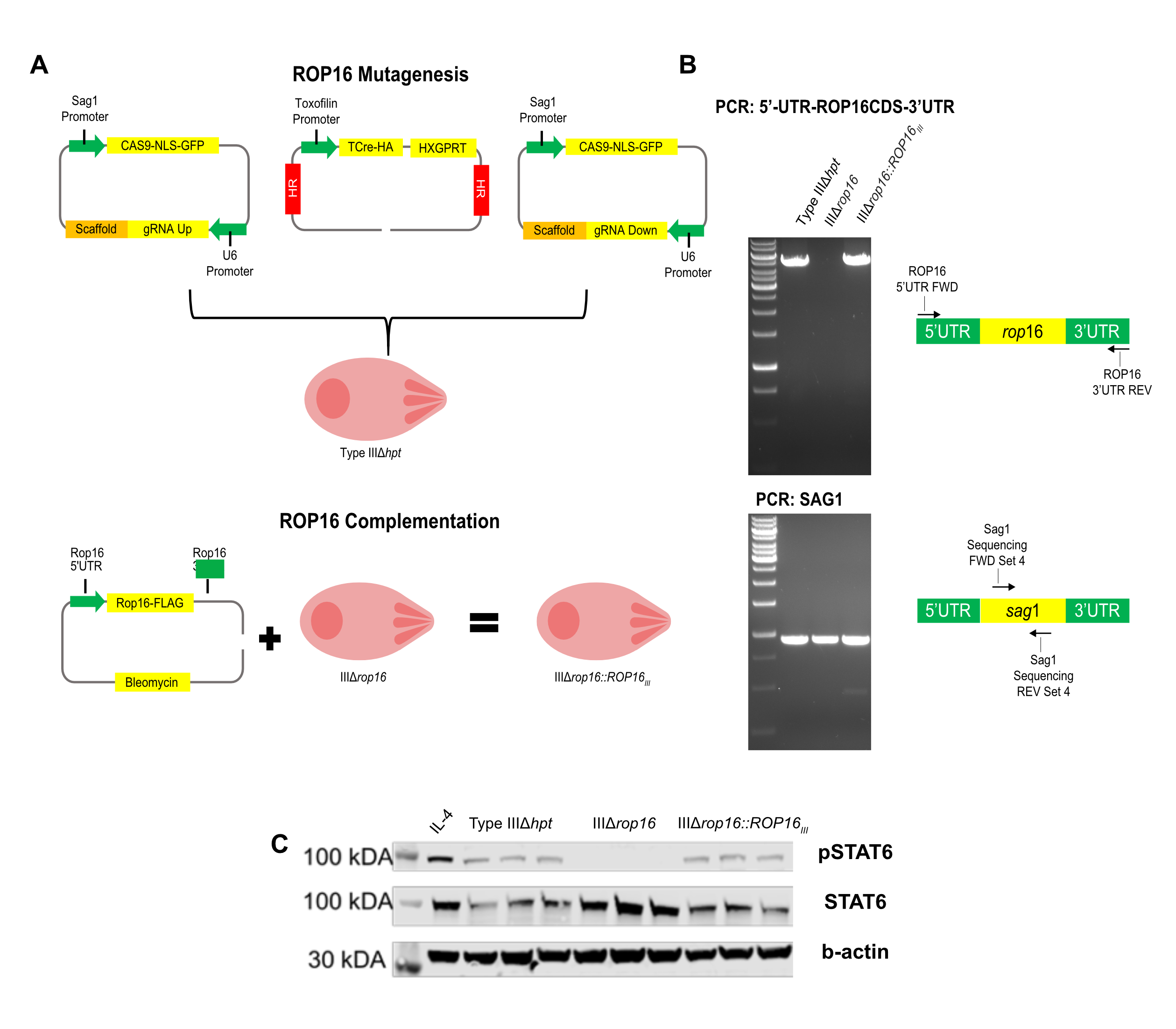

Supplement: S5 Fig — A. Schematic representation of the approach used to create the IIIΔrop16 and IIIΔrop16::ROP16III complemented strains. Type IIIΔhpt parasites were transfected with CRISPR/CAS9 vectors targeting 500bp upstream (gRNA Up) and downstream (gRNA Down) of the rop16 coding sequence and a linearized vector with 500bp regions of homology (HR) to the 5’ and 3’UTRs of rop16 surrounding either the selectable marked HXGPRT alone (not shown) or the selectable marked HXGPRT and the toxofilin-Cre coding sequence (shown). Complementation was achieved using a linearized vector encoding a FLAG-tagged ROP16 and a selectable bleomycin-resistance marker. B. PCR of the entire rop16 locus for the IIIΔrop16 and IIIΔrop16::ROP16III strains. PCR analysis of SAG1 was used as a DNA control. C. Western blots from HFFs stimulated with IL-4 or infected with parental (Type III), IIIΔrop16, or IIIΔrop16::ROP16III parasites. Protein isolation was done at 18 hours post-infection or stimulation. HFFs were infected at a MOI of 5. (TIFF) [file ppat.1007856.s005.tiff]

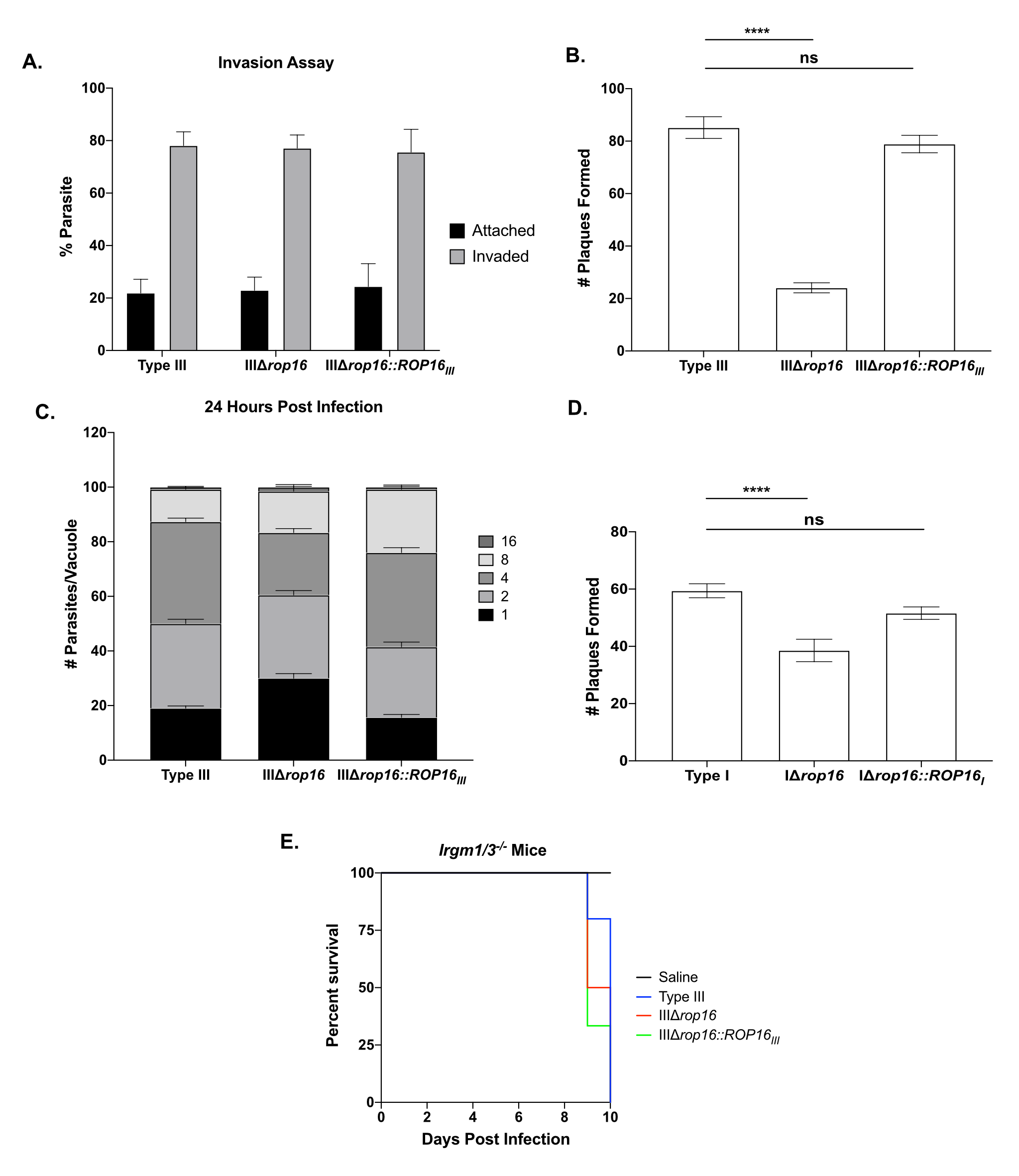

Supplement: S6 Fig — A. Quantification of the percentage of parasites attached versus invaded into human foreskin fibroblasts (HFFs) for Type III (parental), IIIΔrop16, or IIIΔrop16::ROP16III parasites. HFFs were infected at a MOI of 1 with the listed strains and then at 6 hours post infection (hpi) the cultures were stained and assayed for attachment and invasion. Bars = mean ± STD (N = 2 independent experiments, 3 coverslips/experiment, 100 parasites counted/coverslip). B. Quantification of plaques formed on HFF monolayers infected with Type III, IIIΔrop16, or IIIΔrop16::ROP16III parasites. HFF cultures were infected with 250 syringe-lysed parasites of the listed strains and then allowed to grow undisturbed for 10 days. At 10 dpi, the cultures were fixed, stained with Crystal Violet, and then analyzed by light microscopy. Bars = mean number of plaques ± SEM, N = 3 independent experiments, 3 replicates/experiment. C. Quantification of the number of parasites/parasitophorous vacuole at 24 hpi for Type III, IIIΔrop16, or IIIΔrop16::ROP16III parasites. Bars = Mean percentages of parasites per vacuoles ± SEM, N = 3 independent experiments, 3 coverslips/experiment, 100 vacuoles assayed/coverslip. D. Quantification of plaques formed on HFF monolayers infected with Type I, IΔrop16, or IΔrop16::ROP16I. Methodology as in (B) except only 100 syringe-lysed parasites were used per strain. E. Survival curve of Irgm1/3-/- mice intraperitoneally infected with the indicated strains. B, D. **** p ≤ 0.0001, ns: not significant, Dunnet’s multiple comparisons test with each parasite line was analyzed against Type III or Type I, as appropriate. (TIF) [file ppat.1007856.s006.tif]
