# Supplementary material for: The ROP16III-dependent early immune response determines the subacute CNS immune response and type III Toxoplasma gondii survival
Source: PLoS Pathog. 2019 Oct 24;15(10):e1007856. doi: 10.1371/journal.ppat.1007856 (PMC6812932; doi:10.1371/journal.ppat.1007856)
Supplement: S1 Table — The table shows the mean concentration (pg/ml) ± SEM of cytokines and chemokines. Blue represents those cytokines or chemokines with a ≥2-fold change over saline treated controls. p-values are based on one-way ANOVA with Bonferroni post-hoc test. (DOCX) [file ppat.1007856.s007.docx]

| **Cytokine/Chemokine** | **Control (Uninfected)** | **Type II** | **Type III** | **p-value (Type II vs. Type III)** |
| --- | --- | --- | --- | --- |
| **CCL5 (RANTES)** | 0.34+0.04 | 45.78+11.12 | 82.06+6.85 | * |
|  |  |  |  | 0.040 |
| **IP-10** | 19.34+1.56 | 679.87+123.28 | 1003.77+45.08 | ns |
|  |  |  |  | 0.075 |
| **IFN-γ** | 2.86+0.26 | 55.52+20.71 | 157.72+22.40 | * |
|  |  |  |  | 0.012 |
| **MCP1** | 2.35+0.24 | 34.11+6.54 | 93.29+15.62 | ** |
|  |  |  |  | 0.009 |
| **TNF-a** | 0.64+0.07 | 4.33+0.62 | 7.55+0.83 | * |
|  |  |  |  | 0.020 |
| **IL-6** | 1.54+0.04 | 18.45+7.62 | 38.19+6.45 | ns |
|  |  |  |  | 0.196 |
| **KC** | 1.06+0.02 | 6.56+1.08 | 13.69+1.94 | * |
|  |  |  |  | 0.162 |
| **MIP1b** | 1.73+0.06 | 8.04+1.15 | 16.89+3.02 | * |
|  |  |  |  | 0.043 |
| **IL-5** | 2.39+0.22 | 13.55+1.45 | 22.39+1.6 | ** |
|  |  |  |  | 0.002 |
| **IL12 (p40)** | 1.18+0.11 | 5.37+0.63 | 7.73+0.46 | * |
|  |  |  |  | 0.024 |
| **IL-17** | 0.35+0.02 | 1.48+0.16 | 2.34+0.25 | * |
|  |  |  |  | 0.308 |
| **G-CSF** | 3.04+0.09 | 12.79+1.5 | 23.13+1.93 | ** |
|  |  |  |  | 0.002 |
| **IL-1b** | 1.80+0.20 | 6.63+0.79 | 12.35+1.82 | * |
|  |  |  |  | 0.033 |
| **IL-1a** | 2.47+0.45 | 8.86+1.42 | 16.51+2.28 | * |
|  |  |  |  | 0.035 |
| **IL-12 (p70)** | 0.48+0.06 | 1.69+0.19 | 2.64+0.22 | * |
|  |  |  |  | 0.015 |
| **MIP2** | 3.60+0.42 | 8.69+0.53 | 13.06+1.16 | * |
|  |  |  |  | 0.011 |
| **GM-CSF** | 5.55+1.12 | 13.49+0.48 | 15.27+0.39 | ns |
|  |  |  |  | 0.073 |
| **IL-7** | 0.34+0.03 | 0.73+0.04 | 0.87+0.04 | ns |
|  |  |  |  | 0.089 |
| **IL-10** | 2.38+0.14 | 5.17+0.26 | 6.07+0.35 | ns |
|  |  |  |  | 0.177 |
| **IL-13** | 14.02+2.30 | 23.09+1.71 | 24.22+1.16 | ns |
|  |  |  |  | >0.999 |
| **MIP1a** | 5.36+0.99 | 4.74+0.37 | 7.32+0.98 | ns |
|  |  |  |  | 0.102 |
| **IL-4** | 0.29+0.02 | 0.29+0.02 | 0.40+0.02 | ** |
|  |  |  |  | 0.008 |
| **IL-9** | 25.17+2.80 | 37.61+1.52 | 32.75+1.65 | ns |
|  |  |  |  | 0.202 |
| **IL-2** | 2.90+0.85 | 4.33+0.21 | 2.86+0.14 | ** |
|  |  |  |  | 0.006 |
| **IL-15** | 32.10+11.60 | 11.82+0.87 | 12.85+0.91 | ns |
|  |  |  |  | >0.999 |

**Table S1.** **List of cytokines and chemokines from the 25-plex LUMINEX assay.** The table shows the mean pg/ml concentration + SEM of cytokines and chemokines. Blue represents those cytokines or chemokines with a >2-fold change over saline treated controls. p-value based on one-way ANOVA with Bonferroni post-hoc test.
