# Supplementary material for: The ROP16III-dependent early immune response determines the subacute CNS immune response and type III Toxoplasma gondii survival
Source: PLoS Pathog. 2019 Oct 24;15(10):e1007856. doi: 10.1371/journal.ppat.1007856 (PMC6812932; doi:10.1371/journal.ppat.1007856)
Supplement: S2 Table — (DOCX) [file ppat.1007856.s008.docx]

**Table S2: List of cells types characterized between type II and type III-infected mice.**

| **Cell type (Brain)** | **Type II** | **Type III** |
| --- | --- | --- |
| **CD3 T cells** | 1.06+0.2x10^5^ | 1.07+0.2x10^6^ |
| **CD4 (CD3^+^, CD4^+^) T cells** | 7.4+1.7x10^5^ | 6.4+0.97x10^5^ |
| **Activated CD4 (CD3^+^, CD4^+^, CD44^+^) T cells** | 6.9+1.6x10^5^ | 6.1+0.9x10^5^ |
| **CD8 (CD3^+^, CD8^+^) T cells** | 3.2+0.7x10^5^ | 3.1+0.4x10^5^ |
| **Activated CD8 (CD3^+^, CD8^+^, CD44^+^) T cells** | 2.9+0.8x10^5^ | 2.9+0.4x10^5^ |
| **Exhausted (CD3^+^, CD8^+^, PD-1^+^) T cells** | 1.8+0.4x10^4^ | 1.6+0.2x10^4^ |
| **Macrophages (CD45^+^, F4/80^+^, CD11b^hi^, CD11c^low/int^)** | 1.8+0.4x10^5^ | 2.4+0.3x10^5^ |
|  | | |
| **Cell type (Spleen)** | **Type II** | **Type III** |
| **CD3 T cells** | 1.2+0.08x10^7^ | 1.2+0.2x10^7^ |
| **CD4 (CD3^+^, CD4^+^) T cells** | 4.5+0.5x10^6^ | 4.6+0.7x10^6^ |
| **Activated CD4 (CD3^+^, CD4^+^, CD44^+^) T cells** | 2.3+0.2x10^6^ | 2.09+0.3x10^6^ |
| **CD8 (CD3^+^, CD8^+^) T cells** | 5.9+0.4x10^6^ | 5.8+0.8x10^6^ |
| **Activated CD8 (CD3^+^, CD8^+^, CD44^+^) T cells** | 3.3+0.3x10^6^ | 2.9+0.3x10^6^ |
| **Exhausted (CD3^+^, CD8^+^, PD-1^+^) T cells** | 5.7+0.8x10^5^ | 8.4+1.2x10^5^ |
| **Macrophages (CD45^+^, F4/80^+^, CD11b^hi^, CD11c^low/int^)** | 1.5+0.4x10^6^ | 8.4+2.4x10^5^ |
