# Supplementary material for: The ROP16III-dependent early immune response determines the subacute CNS immune response and type III Toxoplasma gondii survival
Source: PLoS Pathog. 2019 Oct 24;15(10):e1007856. doi: 10.1371/journal.ppat.1007856 (PMC6812932; doi:10.1371/journal.ppat.1007856)
Supplement: S3 Table — (DOCX) [file ppat.1007856.s009.docx]

**Table S3. List of Primers used throughout the paper.**

| **Primers** | **Sequences** |
| --- | --- |
| B1 FWD | tcc cct ctg ctg gcg aaa agt |
| B1 REV | agc gtt cgt ggt caa cta tcg att g |
| GAPDH FWD | agg tcg gtg tga acg gat ttg |
| GAPDH REV | tgt aga cca tgt agt tga ggt ca |
| Arg1 FWD | tgg ctt taa cct tgg ctt gct |
| Arg1 REV | aaa gaa caa gcc ctt ggg agg |
| IL-4 FWD | cca tat cca cgg atg cga ca |
| IL-4 REV | aag ccc gaa aga gtc tct gc |
| IL-12 FWD | cag aag cta acc atc tcc tgg ttt g |
| IL-12 REV | tcc gga gta att tgg tgc ttc aca c |
| iNOS FWD | gac ctg atg ttg cca ttg ttg |
| iNOS REV | gat cca gtg gtc caa cct g |
| SagI Sequencing FWD Set 4 | cgc tgc acc act tca tta ttt |
| SagI Sequencing REV Set 4 | tgt tcc cgc aga cga ttt |
| Rop16 gRNA Upstream FWD | aag ttg tgt tgt cgg ttc ccg aat ag |
| Rop16 gRNA Upstream REV | aaa act att cgg gaa ccg aca aca ca |
| Rop16 gRNA Downstream FWD | aag ttg agt tac ttc tca tct cac tg |
| Rop16 gRNA Downstream REV | aaa aca gtg aga tga gaa gta act ca |
| Rop16 Upstream Flank KpnI FWD | gat cag gta cca gtt gtg tct gct tgg aca cg |
| Rop16 Upstream Flank KpnI REV | gat cag gta cct ctt gcg aca aac aag atc ac |
| Rop16 Downstream Flank HindIII FWD | gat caa agc ttg ggt gta agg ttc cca cct t |
| Rop16 Downstream Flank HindIII REV | gat caa agc ttg tga cag gaa cct gcg cc |
| Rop16 Internal Fragment PCR FWD | gga gtt gga tgt tcg gga taa g |
| Rop16 Internal Fragment PCR REV | gct ctt gag ggt ctg gat tta g |
| Rop16 5’UTR FWD | aca aag acg atg acg aca agt agg gtg taa ggt tcc cac c |
| Rop16 5’UTR REV | cgt cat cgt ctt tgt agt cca tcc gat gtg aag aaa gtt cgg |
